# Transcriptomic Changes of Photoperiodic Response in the Hypothalamus Were Identified in Ovariectomized and Estradiol-Treated Sheep

**DOI:** 10.3389/fmolb.2022.848144

**Published:** 2022-04-11

**Authors:** Xiaoyun He, Ran Di, Xiaofei Guo, Xiaohan Cao, Mei Zhou, Xiaoyu Li, Qing Xia, Xiangyu Wang, Jinlong Zhang, Xiaosheng Zhang, Qiuyue Liu, Mingxing Chu

**Affiliations:** ^1^ Key Laboratory of Animal Genetics and Breeding and Reproduction of Ministry of Agriculture and Rural Affairs, Institute of Animal Sciences, Chinese Academy of Agricultural Sciences, Beijing, China; ^2^ Institute of Animal Husbandry and Veterinary Medicine, Tianjin Academy of Agricultural Sciences, Tianjin, China

**Keywords:** hypothalamus, photoperiodic response, hormone, pathways, mRNAs, lncRNAs

## Abstract

Accurate timing of seasonal changes is an essential ability for an animal’s survival, and the change in the photoperiod is the key factor affecting reproductive seasonality in mammals. Emerging evidence has suggested that multiple hypothalamic genes participate in the photoperiod-induced regulation of reproductive activities in sheep, but the mechanism is still unclear. In this study, we initially examined the plasma level of two major reproductive hormones, namely, follicle-stimulating hormone (FSH) and prolactin (PRL), under different photoperiods in ovariectomized and estradiol-treated (OVX + E_2_) sheep using radioimmunoassay (RIA). Of the two hormones, the concentration of PRL significantly increased with the extension of the photoperiod, while FSH showed the opposite trend. Subsequently, an examination of the transcriptomic variation between the short photoperiod (SP) and long photoperiod (LP) was conducted. Differential expression analyses and functional annotation showed that several key genes in the insulin secretion (*VAMP2*, *PRKACB*, *PRKCG*, and *PLCB1*), GnRH (*MAPK13*, *CGA*, *CDC42*, *ATF4*, and *LHB*) pathways, and circadian entrainment (*KCNJ5*, *PER1*, *GNB2*, *MTNR1A*, and *RASD1*), as well as numerous lncRNAs, including XR_173257.3, XR_173415.3, XR_001435315.1, XR_001024596.2, and XR_001023464.2, were shown potentially vital for the hypothalamic photoperiodic response. Four of the differentially expressed mRNAs and lncRNAs were validated by qPCR. The constructed mRNA–mRNA interaction networks further revealed that transcripts potentially participated in hypothalamic thyroid hormone synthesis, endocrine resistance, and neuroactive ligand–receptor interactions. The interactome analysis of lncRNAs and their targets implied that XR_173257.3 and its target arylalkylamine N-acetyltransferase (*AANAT*) and XR_173415.3 and its target *TH* might participate in the regulation of seasonal reproduction. Together, the changes in reproductive hormones and transcriptome will help to determine the important photoperiod-induced lncRNAs and mRNAs and provide a valuable resource for further research on reproductive seasonality in sheep.

## Introduction

Seasonal animals can use changes in the photoperiod to guide seasonal reproduction to ensure the survival of the next generation. Several studies have demonstrated that most mammals have a highly accurate mechanism for photoperiod measurement and show dramatic changes in response to small photoperiod changes (Nishiwaki-Ohkawa and Yoshimura, 2016; Nakayama and Yoshimura, 2018). The annual cycles of mammalian reproductive activities are controlled by the hypothalamic–pituitary–gonadal axis (HPGA), which plays a vital role in reproduction in mammals from fetal development through puberty to sexual maturity (Plant, 2015; Gao, et al., 2018; Hill and Elias, 2018). In mammals, the eye is believed to be the only photoperiod receptor, and the light received by the eyes is transmitted to the pineal gland through the suprachiasmatic nucleus (SCN) and causes melatonin secretion (Lincoln, et al., 2006). As the target of melatonin and an important functional region in the brain, the mediobasal hypothalamus (MBH) can release gonadotropin-releasing hormone (GnRH) to activate the secretion of gonadotropins (luteinizing hormone (LH) and follicle-stimulating hormone (FSH)) and initiate a series of estrus and reproductive activities. In these processes, the change in the photoperiod is the original and key factor affecting hypothalamic function. As previously reported, melatonin can alter hundreds of genes in mammals, including the seasonal expression of thyroid-stimulating hormone β (TSHB) in the pars tuberalis (PT) and MBH, by targeting a special population of PT cells (Wood, et al., 2015; Lomet, et al., 2018). TSH in the PT connects melatonin with hypothalamic triiodothyronine (T3) *via* the variation of Dio2 concentration in the third ventricle, which may be crucial for photoperiod-induced seasonal timing (Wood and Loudon, 2014; Helfer, et al., 2019). Numerous gene expression patterns in the MBH which are associated with the initiation of photoperiod-induced secretion of LH were also studied in Japanese quail (Nakao, et al., 2008). In addition, insulin signaling was also widely detected in the hypothalamus, and the interruption of insulin signaling can lead to abnormalities in puberty in mice, which has been suggested to be related with puberty (Burcelin, et al., 2003; Qiu, et al., 2013). Moreover, a population of Kiss1 neurons in the arcuate nucleus (ARC) is also indispensable in seasonal reproduction in hamster and sheep (Smith, 2012; Harter, et al., 2018; He, et al., 2018).

In recent years, numerous lncRNAs have been found to play a crucial role in the reproductive regulation in mice (Taylor, et al., 2015), goats (Gao, et al., 2018), rats (Gao, et al., 2018), and sheep (Zhang, et al., 2019; Chen, et al., 2021). The hypothalamus plays an important role of the transmission center in the seasonal reproduction of animals. The role of lncRNAs in modulating the function of the hypothalamus is also well documented. For example, a novel lncRNA, GnRH1 enhancer-derived non-coding RNA (GnRH-E1 RNA), is expressed in the hypothalamus in both mice and rats, and the knockdown of GnRH-E1 RNA resulted in a significant decrease in the expression of GnRH1 in mice (Huang, et al., 2016). A previous study showed that RMST, a novel lncRNA, can direct binding to the transcription factor Sox2 to participate in the ontogeny of GnRH neurons and puberty in a Kallmann syndrome (KS) patient (Stamou, et al., 2019). In addition, our earlier hypothalamic studies found that several candidate lncRNAs, such as MSTRG.26777, MSTRG.105228, and MSTRG.95128, may regulate ovine reproduction by targeting nearby genes (Zhang, et al., 2019). Although the function of lncRNA in the hypothalamus has rarely been studied, we can speculate that it is important to further explore the function of the ovine hypothalamus.

To date, our understanding of how photoperiod induced the changes of the molecular neuroendocrine axis and reproductive seasonality remains limited, the majority of studies in quail, hamsters, and sheep focused on the comparison of differences in long and short photoperiods (LP vs. SP) (16 vs. 8 h light), such as the change of hormonal concentration and key gene or protein expression (Dardente, et al., 2019; Guh, et al., 2019). We lack a comprehensive view of the impact of increasing daylengths on the transcriptome level within the hypothalamus. In the present study, we established an animal model for sheep estrus research based on the ovariectomized (OVX), which has been used for the functional study of the mammalian hypothalamus in rats, mice, Siberian hamster, goats, and sheep (Lomet, et al., 2018). In this model, the serum levels of FSH and PRL can provide reliable information of photoperiodic change. On the other hand, high-throughput sequencing was used to investigate the transcriptomic changes in the hypothalamus. The findings will provide some new information for understanding the genetic basis and molecular mechanisms of the hypothalamus in ovine seasonal reproduction.

## Materials and Methods

### Animals and Sample Collection

**FIGURE 1:**
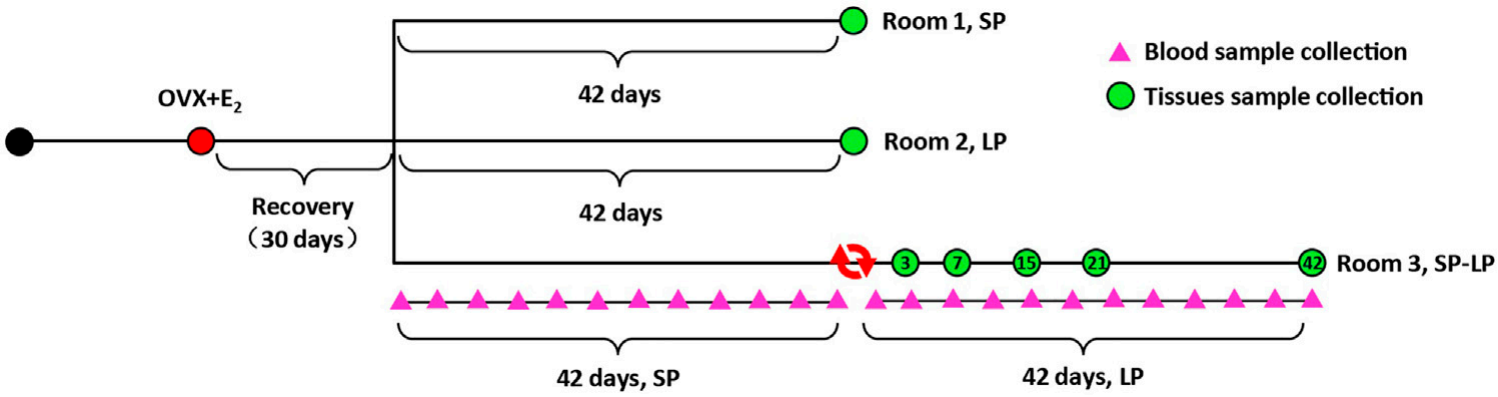
Hormone preparation and sample collection timeline. All OVX + E_2_ ewes were assigned to three experimental groups in three rooms: short photoperiod (SP), long photoperiod (LP), and short photoperiod transfer to long photoperiod (SP-LP). A total of 24 pink triangles represent the day of blood sample collection in SP-LP groups, and seven green circles were the time of hypothalamus sample collection. In addition, the tissue and blood samples were collected at 8 a.m. and 2 p.m., respectively.

A group of 21 Sunite ewes (35–40 kg, 3 y, clinically normal, and non-pregnant) were selected from Urat Middle Banner, Bayan Nur City, Inner Mongolia Autonomous Region, China and housed in a farm at Tianjin Institute of Animal Sciences, Tianjin (39°N latitude), China. All ewes were fed *ad libitum* and had free access to water. OVX + E_2_ sheep and light control rooms were constructed as previously described (La, et al., 2020; He et al., 2021; Xia, et al., 2021). In brief, estradiol treatment was achieved with an inner diameter of 3.35 mm and an outer diameter of 4.65 mm, packed with 20 mg crystalline 17β-estradiol (Sigma Chemical Co., St. Louis, MO). Implants were inserted into the axillary region and designed to produce the circulated E_2_ levels of approximately 3–5 pg/ml for 2 weeks (Smith, et al., 2007). Then, all ewes were maintained in one of three photoperiod-controlled rooms, SP: short photoperiod (8/16 h light–dark), LP: long photoperiod (8/16 h light–dark) and SP–LP: photoperiod from short transfer to long). Along with the photoperiodic treatments, slaughter was performed on SP42 or LP42, SP-LP3 (the third day of SP–LP), SP-LP7, SP-LP15, SP-LP21, and SP-LP42 (n = 3). After slaughtering, the hypothalamus was rapidly removed from the brain, flushed with PBS (pH 7.4), snap-frozen at liquid-nitrogen, and stored at −80°C for subsequent study. Simultaneously, jugular venous blood samples were collected twice weekly (24 times, 12 times for different photoperiods) from three ewes in the SP–LP room. After centrifugation, serum was collected and frozen until assayed for hormones. The hormone preparation and sample collection’s schematic diagrams are shown in Figure 1.

### Hormone Assays

The iodine (^125^I) human follicle-stimulating hormone radioimmunoassay kit and the iodine (^125^I) prolactin radioimmunoassay kit (Beijing North Biotechnology Research Institute Co., Ltd., Beijing, China) were used for FSH and PRL assays by radioimmunoassay (RIA). All samples from different time points were measured in triplicate. The FSH kit had an assay sensitivity of <1.0 mIU/ml, and the PRL kit sensitivity was ≤40 uIU/ml.

### RNA Isolation and Library Preparation and Sequencing

Total RNA was isolated from each sample using TRIzol reagent (Invitrogen, Carlsbad, CA, United States), and 1% agarose gels were used to detect the degradation and contamination prior to subsequent. In addition, RNA’s purity, concentration, and integrity were also detected by a NanoPhotometer^®^ spectrophotometer (IMPLEN, CA, United States), a Qubit^®^ RNA Assay Kit in Qubit^®^ 2.0 Fluorometer (Life Technologies, CA, United States), and the RNA Nano 6000 Assay Kit of the Bioanalyzer 2100 system (Agilent Technologies, CA, United States), respectively. Subsequently, libraries were generated using rRNA-depleted RNA with the NEBNext^®^ Ultra™ Directional RNA Library Prep Kit for Illumina^®^ (NEB, Ipswich, MA, United States) according to the manufacturer’s recommendations. Finally, the libraries were sequenced on an Illumina HiSeq 4000 platform, and 150 bp paired-end reads were generated.

### Quality Control and Bioinformatics Analysis of Sequenced RNAs

Clean data were obtained by removing reads-containing adapter, reads-containing poly-N sequence, and low-quality reads from raw data. Simultaneously, the Q20, Q30, and GC contents of the clean data were calculated. All the following analyses were based on the clean data. The *Ovis aries* reference genome and gene model annotation files were downloaded from the genome website directly (Oar_v4.0). The index of the reference genome was built using bowtie2 (v2.2.8) (Sirén, et al., 2014). Reads were aligned to the reference genome using HISAT2 (v2.0.4). The mapped reads of each sample were assembled by StringTie (v1.3.1) (Pertea, et al., 2016).

To improve the statistical validity of the identification, a series of strict screening conditions were employed to identify lncRNAs. First, we removed transcripts that had fewer than two exons, and transcripts of less than 200 bp in length were discarded. The remaining transcripts were BLAST to known sheep lncRNAs using Cuffcompare, and lncRNAs that overlapped with the transcripts in the database were incorporated into the subsequent analysis. Next, transcripts with an FPKM ≥ 0.5 were retained after calculating the expression of each transcript by Cuffdiff. Finally, CNCI (Sun, et al., 2013), CPC (Kong, et al., 2007), and Pfam (El-Gebali, et al., 2019), three databases were used to identify the candidate lncRNAs. The intersections of the results from each program were defined as the novel lncRNA transcripts, and those identified by all three tools were defined as the final candidate lncRNAs for further analysis.

### Differential Expression Analysis and Validation of Sequencing Data by qPCR

Cuffdiff (v2.1.1) was used to calculate FPKMs, which represent the gene expression level of both the lncRNAs and coding genes in each sample (Trapnell, et al., 2010). FPKMs were computed by summing the FPKMs of transcripts in each gene group. A criterion of absolute log2 (fold change) > 1 and *p* ≤ 0.05 between the two groups was used to identify differentially expressed genes in Cuffdiff.

Eight transcripts (four mRNAs and four lncRNAs) were selected for each group to validate the RNA-sequencing results by qRT-PCR. The specific primers are listed in Table 1 cDNA was synthesized from the RNA samples remaining after sequencing with the PrimeScript™ RT reagent Kit (TaKaRa, Dalian, China). qPCR was performed with a SYBR Green assay (TaKaRa, Dalian, China) on a Roche LightCycler 480 (Roche Applied Science, Mannheim, Germany). The qPCR mixture and program has been described in our previous study (Zhang, et al., 2019). All samples were examined in triplicate. Differentially expressed mRNA and lncRNA levels were normalized to β-actin to determine the relative expression using the 2^−ΔΔCt^ method (Livak and Schmittgen, 2000).

**TABLE 1:** Primers of the validation of differentially expressed mRNAs and lncRNAs.

Transcript type	Transcript name	Forward primers	Reverse primers	Product size
*mRNA*	PLCB1	GAA​CCT​AAC​AAC​AGC​CTC​GC	AGT​GAG​AAA​GGG​GCT​GAG​AC	139
	PRKACB	ACG​GTT​CTA​TGC​AGC​TCA​GA	GGC​AAA​CCC​AAA​GTC​TGT​GA	136
	SMAD4	CAG​CAC​CAC​CAA​TTT​TCC​CA	GGT​GCA​GTC​CTA​CTT​CCA​GT	198
	BCL2	TCT​TTG​AGT​TCG​GAG​GGG​TC	GGC​CAT​ACA​GCT​CCA​CAA​AG	162
	ATF4	CAG​CAG​CTA​CTA​GGT​ACC​CC	CCT​TGC​TTT​GCG​AAC​CTC​TT	167
	RASD1	CCG​CAA​GTT​CTA​CTG​CAT​CC	CTT​GGT​GTC​GAG​AAT​CTG​CC	193
	CLOCK	ACG​AGA​ACT​TGG​CAT​TGA​A	CTT​CCT​TGA​GAC​TGA​CTG​TAT	151
	CREB1	TTG​CCA​CAT​TAG​CCC​AGG​TA	GCC​GCC​TGA​ATA​ACT​CCA​TG	121
	IGF2	ATG​GGG​ATC​ACA​GCA​GGA​AA	GGA​TGG​TCG​GCT​GAA​GTA​GA	168
	DIO2	GAA​GGA​ATG​CGC​TGC​ATC​TG	GGG​AAT​TGG​GGG​CAT​CTT​CA	82
	BHLHE41	TTG​ACA​ACT​CTG​GGG​CAT​CT	CGC​TCC​CCA​TTC​TGT​AAA​GC	125
	LHB	CCT​GCC​CTG​TCT​GTA​TCA​CT	ACG​GGG​AAG​GAG​ACC​ATT​G	187
	ESR1	TGA​AGT​GCA​AGA​ACG​TGG​TG	CTG​CCT​CCC​CAG​TGA​TGT​AA	183
	GNB2	GAC​GGC​AAG​CTC​ATC​ATC​TG	ACG​GGT​CTT​GAG​GCT​GTA​AA	162
	TSHB	GGC​AAG​CTG​TTT​CTT​CCC​AA	GTA​ACA​TGG​CGT​GGA​CAT​CC	104
	FBXL3	TCC​AAA​TCC​TTG​TCC​TCG​CT	TGA​TCA​GCC​ACA​CAC​AGG​AT	155
	PRL	CCT​GGA​GCC​AAA​GAG​ACT​GA	ATC​TTG​CTT​GAA​TCC​CTG​CG	131
lncRNA	LNC_004953	TTT​TCT​CCT​GGG​GTT​GAG​CA	AAA​CTC​GTC​TCA​AGC​CTC​CA	143
	LNC_002738	GAC​AAC​TAC​TGC​TGG​GGT​CT	CAC​ACC​CCA​AAG​AAG​TCA​GC	184
	LNC_008255	TCC​CAG​CAA​AGG​AGA​CAG​AG	CAC​AAC​CCT​ACC​AAA​GCC​AC	146
	LNC_008321	GGG​CTC​AGT​TCC​ACT​CTT​CT	AGA​GAC​ATG​GCA​GCT​TCC​TT	159
	XR_173415.3	GAT​CGG​TGC​CTT​TGA​GCT​TG	CTC​CAT​CAC​ACC​GGA​CCA​TA	129
	XR_173257.3	AGC​AAG​TGG​GAA​GGT​CTA​CC	CAA​TGG​TTA​GGA​CTC​AGC​GC	168
	XR_001043724.2	CGT​TTG​CAC​TAC​CAC​ACA​CA	ACC​CAA​GAC​GCA​CTG​TAG​AA	165
	XR_001045234.2	AGG​CTA​AGA​GGG​AGG​TCA​GA	TTG​CTG​TGG​TCT​GGA​ATT​GC	153
	XR_001023464.2	GAG​AAA​GTG​GAG​TCC​GAG​CT	TCA​CCA​CAA​GCA​ACT​TCA​GC	158
	XR_001020627.1	GGT​CAA​GAA​TCC​ACC​TCC​CA	GGC​CCT​AGG​TTG​GAA​GTC​TT	122
	XR_001034881.2	TTC​TCC​TTC​CCG​TGT​CTC​AC	TCG​TCC​AGT​CAG​CAC​TCT​TT	143
	XR_001433797.1	CTG​ATC​AAA​TGG​GCC​TTG​GG	AAT​GGC​AGC​ACA​AAT​CAG​CA	120
	XR_001433798.1	AAT​GGC​AGC​ACA​AAT​CAG​CA	CTG​ATC​AAA​TGG​GCC​TTG​GG	120
	XR_001024596.2	TGT​AGA​AAA​GCC​TGG​CGA​GA	CTC​CAT​CCA​CAC​CAT​GTC​CT	143
	XR_001434242.1	GCC​CTT​TTC​TCC​CTT​CTC​CT	TCT​GGC​CAC​GTT​TCT​GTT​TG	117
	XR_001027187.1	GCT​GCT​GTT​AGA​AGA​ACC​GG	GGA​GGA​AGT​AGT​TGT​GGG​CT	84
	XR_001039609.1	TGA​TTG​CTC​ACC​TGT​TCC​CT	CTC​CAC​ACA​TCA​CTC​CCA​GT	153
	XR_001023520.1	TGC​ACC​CCA​CTG​ATC​AGA​TT	AAG​TCA​TCA​GGT​CTG​CTG​CT	181
	XR_001434385.1	TGC​TCA​CCC​ACT​TCT​CCA​TT	TTA​GCT​CCC​AAC​TTC​GGT​GT	154

### lncRNA Target Gene Prediction and Functional Annotation and Enrichment Analysis

To further elucidate the functions of DE-mRNAs and DE-lncRNAs, we predicted the target genes of the lncRNAs in *cis* and *trans*. To understand the functional roles of the target genes of the lncRNAs, we used GOseq R package to implement enrichment analysis. In addition, the differentially expressed protein-coding genes were also analyzed using GO. We employed KOBAS software to detect the enrichment of lncRNA target genes or differentially expressed genes in KEGG pathways. GO terms with -log10 (*p*-value) < 0.05 and KEGG pathways with *p* < 0.05 were considered significantly enriched.

### Construction of mRNA–mRNA and lncRNA–mRNA Networks

To infer the functions of the photoperiod-based DE-lncRNAs and DE-mRNAs in the ovine hypothalamus, a protein–protein interaction (PPI) network between the protein-coding genes was constructed based on information from STRING. Additionally, we constructed a complementary pair network based on mRNA and mRNA as well as between mRNA and lncRNA by using Cytoscape (V3.6.1).

### Statistical Analysis

The statistical evaluation of the experimental results was performed with Student’s *t*-test using SPSS 20.0 statistical software. All data are expressed as the means with standard error (SE). For hormone profiles, RIA data were analyzed by repeated measures ANOVA, and comparisons of hormones between different photoperiods and qPCR validation were performed by one-way ANOVA. *p* < 0.05 was considered statistically significant.

## Results

### Hormone Profile Analysis

**FIGURE 2:**
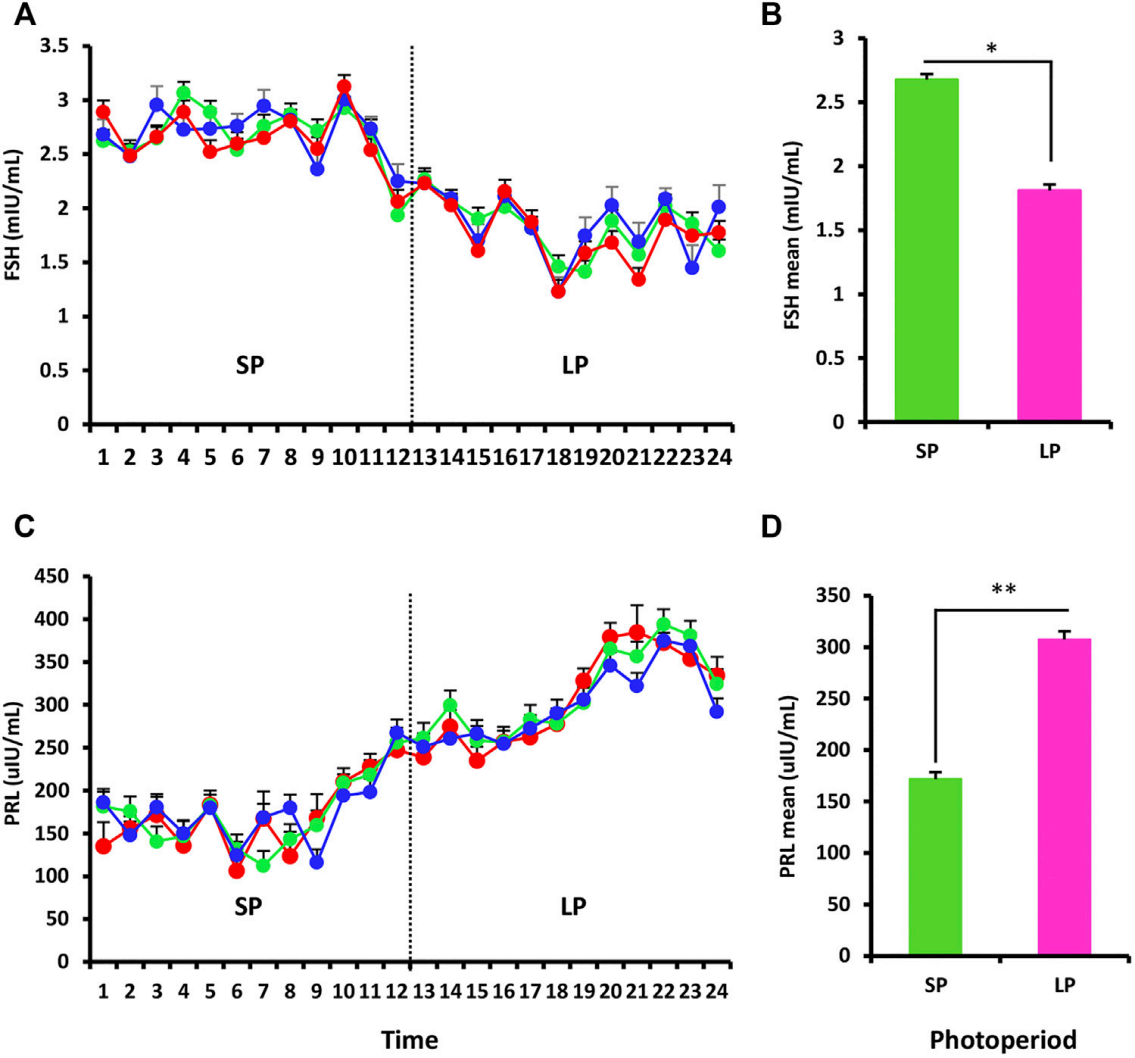
Hormonal profiling of the response to the different photoperiodic treatments. **(A)** Mean follicle-stimulating hormone (FSH) levels in blood serum of ewes sampled twice weekly throughout the experiment. **(B)** Mean of FSH for the SP and LP (12 time points each treatment). **(C,D)** Prolactin (PRL) profiles, the picture interpretation identical to those for FSH. Results were expressed as mean ± SE, * represents *p* < 0.05, ** represents *p* < 0.01, *** represents *p* < 0.001.

RIA was used to assess the serum hormone levels of FSH and PRL in three ewes exposed to the changed photoperiod. The FSH data shown in Figure 2A mostly reflected the impact of the photoperiod, and the serum hormone levels differed significantly between SP and LP (*p* < 0.05) (Figure 2B). For PRL, the concentration increased with the photoperiodic transition from SP to LP (Figure 2C), and the difference also reached significant level (*p* < 0.01) (Figure 2D).

### Summary of RNA-Sequencing Data

**FIGURE 3:**
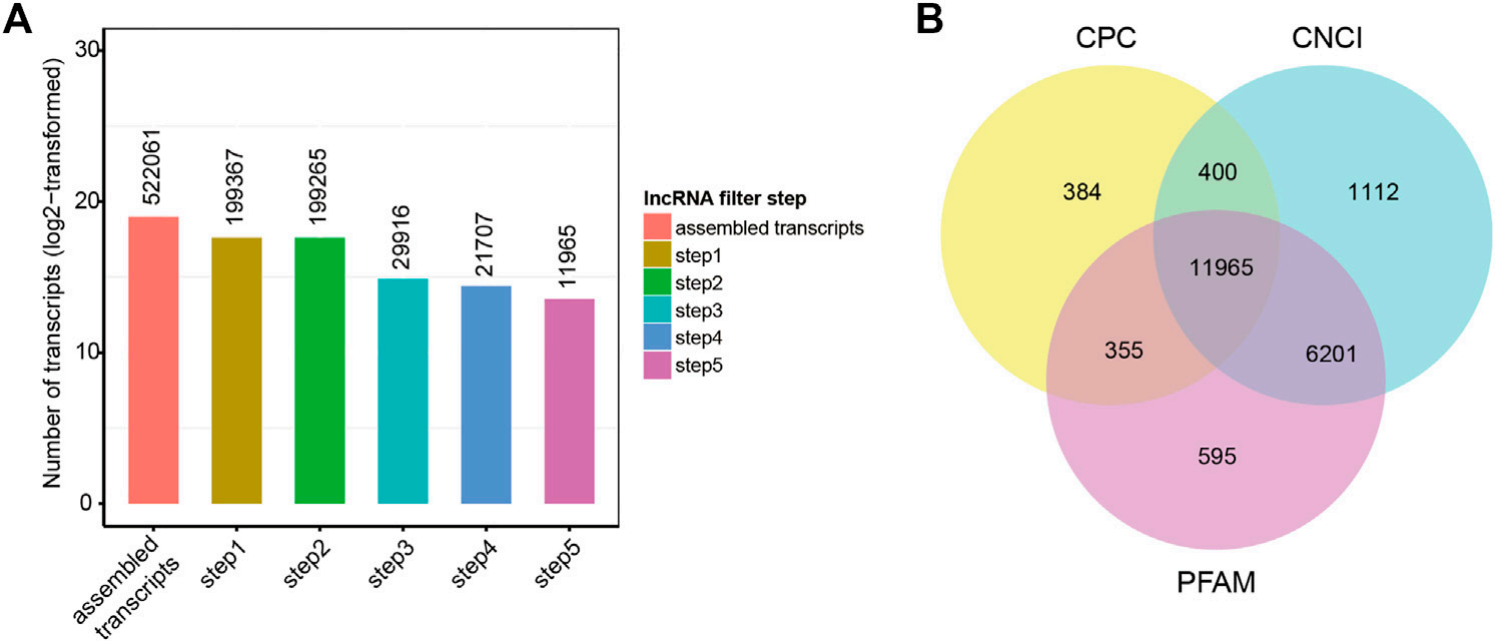
Screening of the candidate lncRNAs in the ovine hypothalamus transcriptome. **(A)** 522,061 transcripts were assembled and 11,965 putative non-coding transcripts were retained for the next analysis after five steps filtering. **(B)** lncRNAs identification was also used in CPC, PFAM, and CNCI.

Twenty-one hypothalamus samples from seven groups (SP42, LP42, SP-LP3, SP-LP7, SP-LP15, SP-LP21, and SP-LP42, n = 3) were used to construct 21 cDNA libraries for sequencing. More than 268 million raw reads were generated for each group. The Q30 of the data was not less than 89%, and the GC contents of each library ranged from 49.34 to 55.81%. In addition, 303.38 Gb clean reads were retained and used in the following analysis after discarding reads with adapters or a poly-N content >10% and other low-quality reads. Approximately, 97% of the clean reads from each library were mapped to the sheep reference genome (Oar_v4.0) (Table 2), and 552,061 transcripts were assembled from these 21 libraries, which were assembled using Scripture and Cufflinks. After a rigorous 5-step screening, 11,965 putative non-coding transcripts were retained (Figure 3A), and the intersection of CNCI, CPC, and Pfam yielded the same result (Figure 3B), identifying 9,645 lincRNAs (80.6%), 2,320 antisense lncRNAs (19.4%), and 0 intronic lncRNAs (0.0%).

**TABLE 2:** Summary of RNA-sequencing data.

Sample name	Raw reads	Clean reads (rate)	Clean bases (G)	Q20 (%)	Q30 (%)	GC content (%)	Total mapped (mapping rate)
SP42a	92,404,972	90,414,754 (97.85%)	13.56	95.87	89.85	51.16	81,762,883 (90.43%)
SP42b	100,053,902	97,725,590 (97.67%)	14.66	95.61	89.34	51.12	88,016,468 (90.06%)
SP42c	91,051,110	88,971,128 (97.72%)	14.67	95.69	89.50	52.08	80,039,501 (89.96%)
LP42a	90,275,678	88,358,798 (97.88%)	13.25	96.01	90.15	51.64	79,497,567 (89.97%)
LP42b	87,254,372	85,217,084 (97.67%)	12.78	95.39	88.97	49.34	76,350,349 (89.60%)
LP42c	90,188,246	88,048,152 (97.63%)	13.21	96.04	90.40	51.00	80,756,426 (91.72%)
SP_LP3a	85,650,368	83,526,432 (97.52%)	14.74	96.16	90.59	52.22	77,200,035 (92.43%)
SP_LP3b	130,883,606	128,480,770(98.16%)	14.75	95.69	89.59	51.41	112,053,275 (87.21%)
SP_LP3c	116,032,590	111,104,312 (95.75%)	14.76	97.09	92.43	51.46	98,109,769 (88.30%)
SP_LP7a	97,605,822	95,353,208 (97.69%)	14.8	96.05	90.38	53.55	87,815,730 (92.10%)
SP_LP7b	103,323,810	100,247,300 (97.02%)	14.81	95.77	89.88	55.14	91,259,752 (91.03%)
SP_LP7c	115,358,562	112,207,154 (97.27%)	14.82	95.65	89.56	54.31	96,346,980 (85.87%)
SP_LP15a	85,180,124	83,403,140 (97.91%)	14.68	95.96	90.08	55.81	74,702,551 (89.57%)
SP_LP15b	92,540,396	89,185,078 (96.37%)	14.69	96.02	90.06	54.23	81,572,860 (91.46%)
SP_LP15c	90,057,266	87,781,282 (97.47%)	14.7	95.82	89.85	52.55	76,214,584 (86.82%)
SP_LP21a	96,002,310	93,740,072 (97.64%)	14.71	95.61	89.48	52.70	80,905,747 (86.31%)
SP_LP21b	98,333,076	96,396,122 (98.03%)	14.72	95.65	89.51	52.97	83,299,568 (86.41%)
SP_LP21c	105,964,310	103,390,136 (97.57%)	14.73	95.52	89.27	53.10	89,946,823 (87.00%)
SP_LP42a	83,425,796	82,323,670 (98.68%)	14.77	95.99	90.27	51.46	71,313,457 (86.63%)
SP_LP42b	96,697,816	94,981,942 (98.23%)	14.78	95.73	89.88	52.67	81,183,909 (85.47%)
SP_LP42c	100,425,940	98,772,632 (98.35%)	14.79	95.43	89.23	54.04	85,854,187 (86.92%)

### Comparison of mRNA and mRNA Characteristics

**FIGURE 4:**
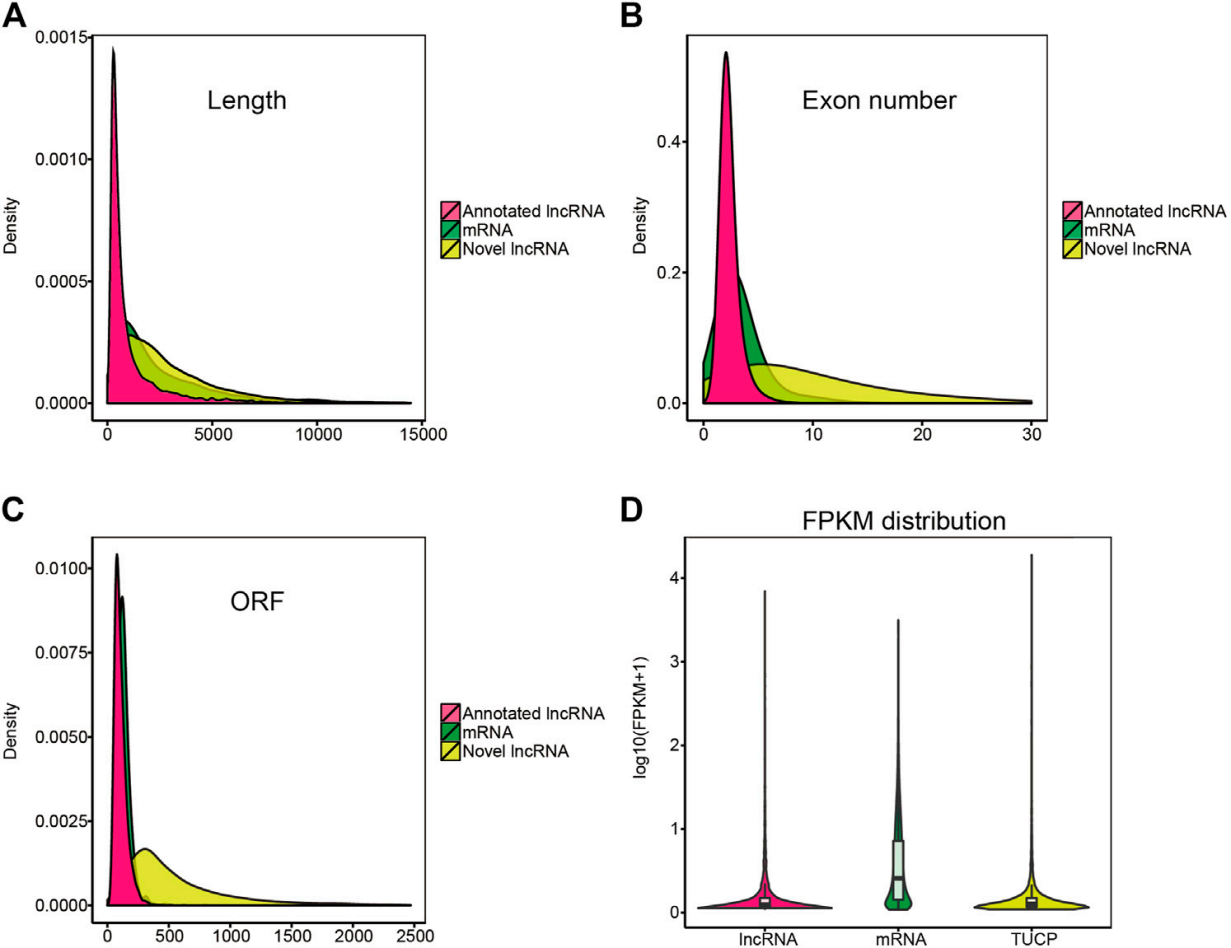
Characterization of candidate lncRNA and mRNA. **(A–C)** Length, exon number, open reading frame (ORF) length distribution of mRNA, annotated lncRNA, and novel lncRNA. **(D)** violin plot of expression level about mRNAs, annotated-lncRNAs, and novel-lncRNAs.

In this study, 13,859 lncRNAs and 44,437 mRNAs were identified. Our results indicated that the length and ORFs of lncRNAs were shorter than those of mRNAs (Figure 4A); the expression of lncRNAs was lower than that of mRNAs (Figure 4D); and lncRNAs tended to contain fewer exons (Figure 4B). These results were consistent with those of our previous studies (Zhang, et al., 2019). In addition, some transcripts of uncertain coding potential (TUCP) were expressed in the hypothalamus (Figure 4C).

### Differential Expression Analysis and qPCR Verification

The criteria of |log2 (fold change) |>1 and *p* ≤ 0.05 were used to identify differentially expressed transcripts. The summary of DE transcripts is shown in Table 3, and the details of the differences in expression can be found in Supplementary Table S1 and Supplementary Table S2. Importantly, the differences between different phases of the photoperiod change need to be verified prior to further analysis. In this study, four DE-mRNAs and four DE-lncRNAs were selected from each compared group to validate the RNA-seq data, and the expression of these RNAs was analyzed by qPCR (Supplementary Figure S1). qPCR results showed that the expression levels of selected DE-mRNAs (*PLCB1*, *PRKACB*, *SMAD4*, *BCL2*, *ATF4*, *RASD1*, *CLOCK*, *CREB1*, *IGF2*, *DI O 2*, *BHLHE41*, *LHB*, *ESR1*, *GNB2*, *TSHB*, *FBXL3*, and *PRL*) from the six compared groups were all significantly different (Supplementary Figure S1A). Similarly, the expression of 19 DE-lncRNAs (LNC_004953, LNC_002738, LNC_008255, LNC_008321, XR_173415.3, XR_173257.3, XR_001043724.2, XR_001045234.2, XR_001023464.2, XR_001020627.1, XR_001034881.2, XR_001433797.1, XR_001433798.1, XR_001024596.2, XR_001434242.1, XR_001027187.1, XR_001039609.1, XR_001023520.1, and XR_001434385.1) also reached the level of significant difference (Supplementary Figure S1B), and the expression patterns of these genes and lncRNAs were consistent with the RNA-seq results.

**TABLE 3:** Summary of differentially expressed lncRNAs and mRNAs.

Compared groups	DE-mRNAs			DE-lncRNAs		
	Total	Up	Down	Total	Up	Down
SP42 vs. LP42	688	359	329	22	15	7
SP42 vs. SP-LP3	737	396	341	23	15	8
SP42 vs. SP-LP7	848	400	448	39	15	24
SP42 vs. SP-LP15	979	521	458	34	12	22
SP42 vs. SP-LP21	1,344	722	622	36	19	17
SP42 vs. SP-LP42	1737	1,139	598	58	32	26

### Functional Annotation of Differentially Expressed lncRNAs and mRNAs in the Hypothalamus

**FIGURE 5:**
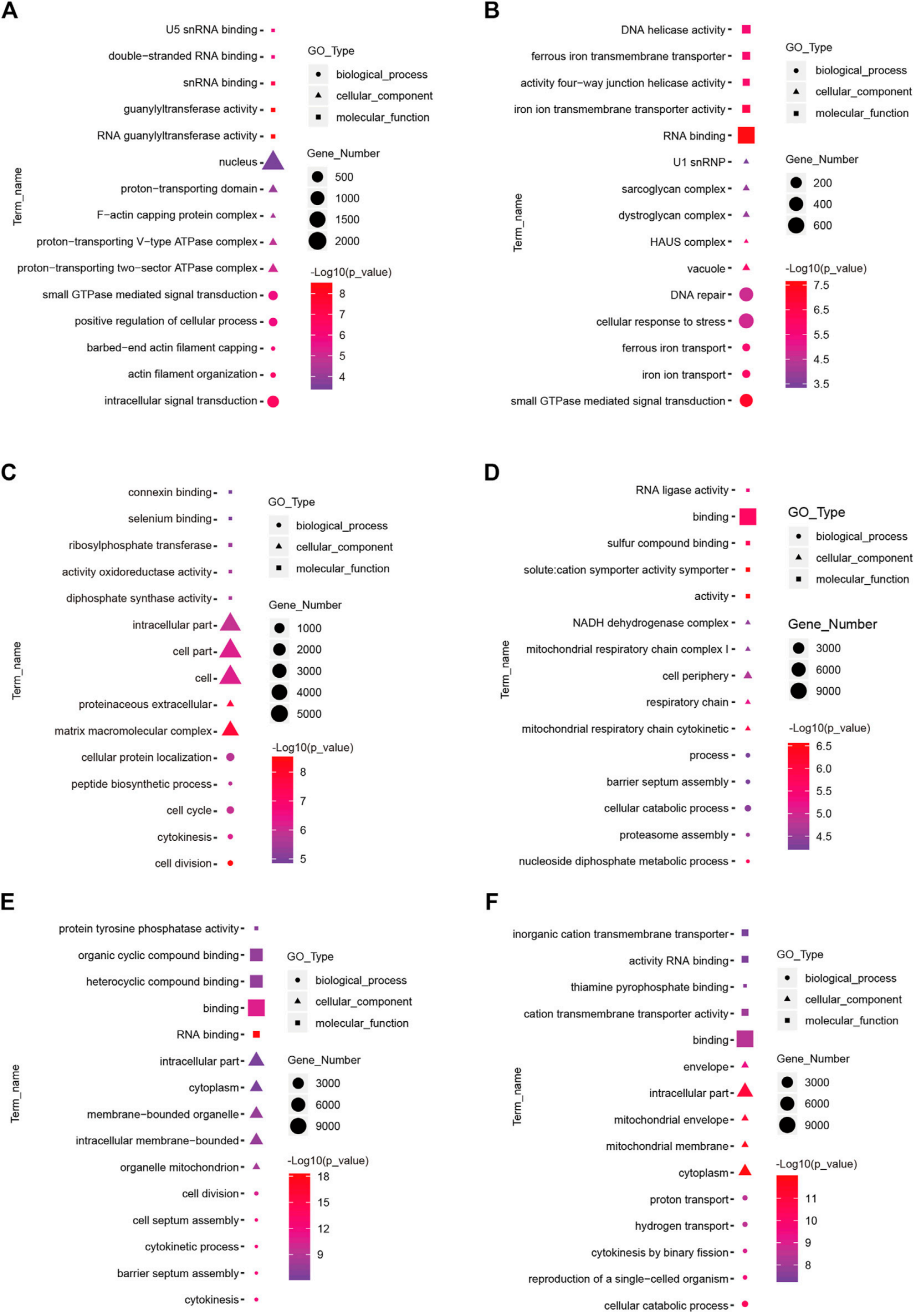
Top enriched Gene Ontology (GO) terms of differentially expressed mRNA between the short photoperiod and long photoperiod in the hypothalamus of Sunite sheep. The GO terms in **(A–F)** are from SP42 vs. LP42, SP42 vs. SP-LP3, SP42 vs. SP-LP7, SP42 vs. SP-LP15, SP42 vs. SP-LP21, and SP42 vs. SP-LP42, respectively.

**FIGURE 6:**
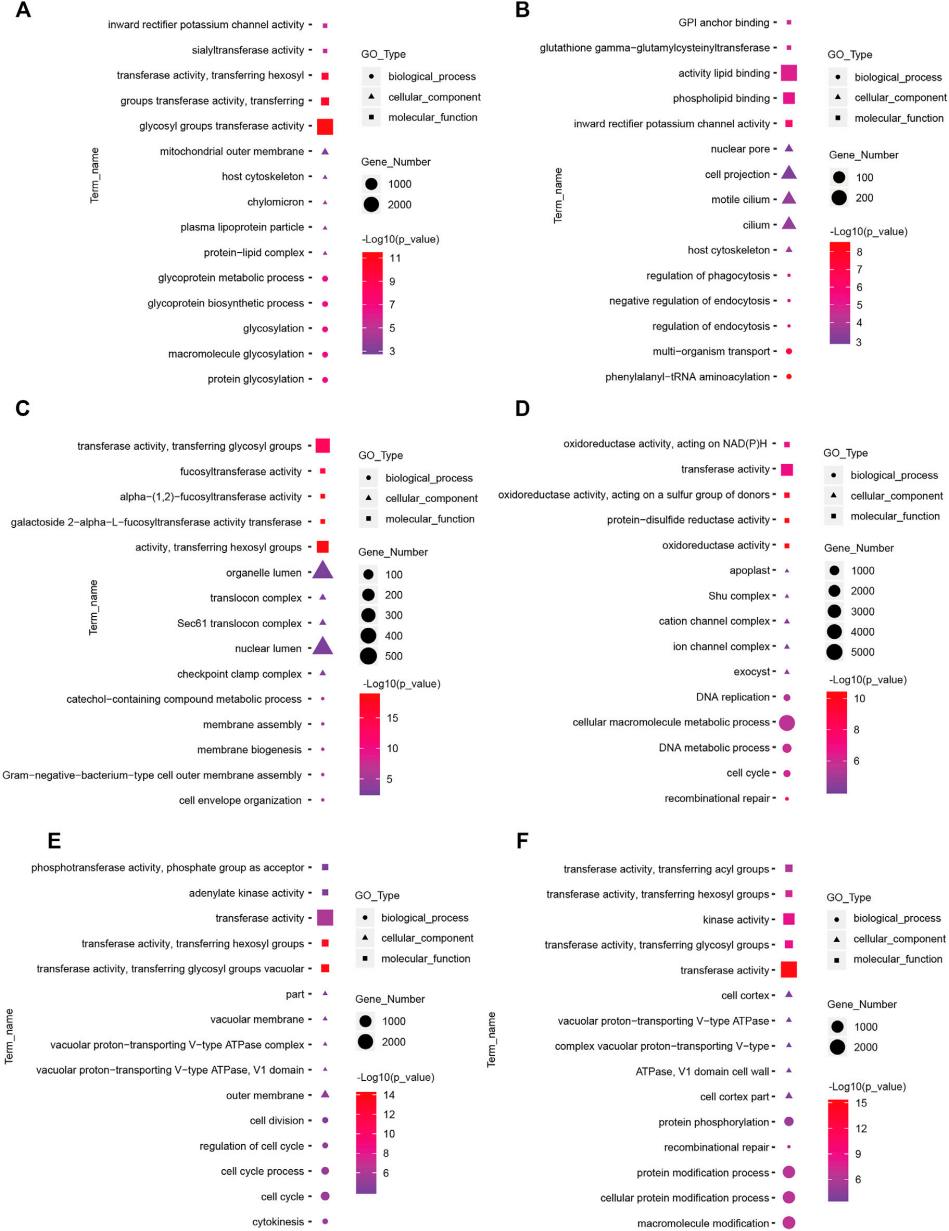
Top enriched Gene Ontology (GO) terms of differentially expressed lncRNAs between the short photoperiod and long photoperiod in the hypothalamus of Sunite sheep. The GO terms in **(A–F)** are from SP42 vs. LP42, SP42 vs. SP-LP3, SP42 vs. SP-LP7, SP42 vs. SP-LP15, SP42 vs. SP-LP21, and SP42 vs. SP-LP42, respectively.

**FIGURE 7:**
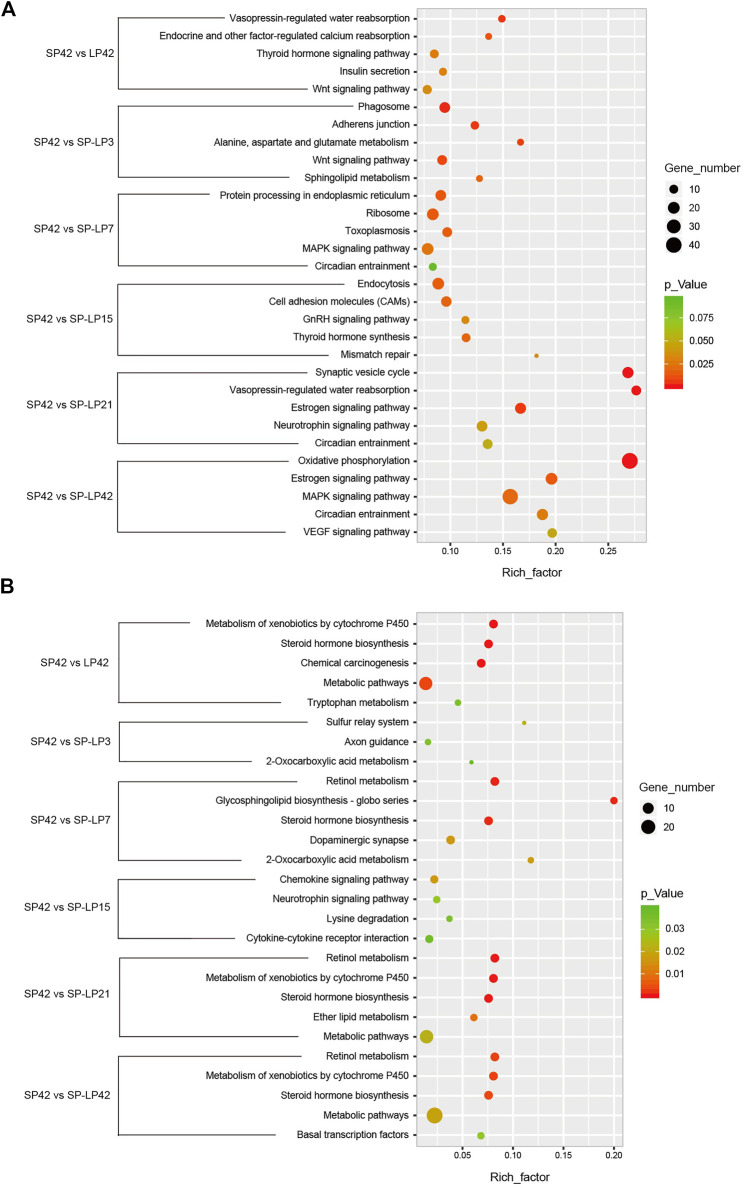
KEGG analysis of differentially expressed mRNAs and lncRNAs targets in hypothalamus about photoperiod responsiveness. **(A)** Five of the top KEGG enrichment pathway for differentially expressed mRNAs; **(B)** three to five of the top KEGG enrichment pathway for differentially expressed lncRNA targets. The longitudinal axis represents the enrichment pathways in different compared groups, and the horizontal axis represents the rich factor of these pathways. The spot size and color represent the number of differentially expressed genes and statistical significance of each pathway, respectively.

To understand the functions of DE-mRNAs and DE-lncRNAs in greater depth, DE-mRNAs and DE-lncRNA-colocalized mRNAs were subjected to GO and KEGG pathway analyses. The top five significantly enriched GO terms were in molecular function (MF), cellular component (CC), and biological process (BP),three GO types for DE-mRNA. Compared with SP42, when the photoperiod changes from SP-LP3 to SP-LP42, blinding, RNA binding, and cell cycle items were significantly enriched (Figure 5, data in Supplementary Table S3). For DE-lncRNA, we used their targets to conduct GO enrichment and most of the significantly enriched GO terms were consistent with mRNAs. In addition, most of the significantly enriched GO terms participate in the regulation of biological and cellular processes (Figure 6, data in Supplementary Table S4). KEGG pathway analysis revealed that these differentially expressed mRNA target genes were enriched in several significant items, such as steroid hormone biosynthesis, dopaminergic synapse, and circadian entrainment (Figure 7A, data in Supplementary Table S5), KEGG pathway analyses of differentially expressed lncRNAs were also enriched in pathways mentioned earlier. In addition, insulin secretion, the thyroid hormone signaling pathway, and the Wnt signaling pathway, were also significantly enriched with the photoperiod changes (Figure 7B, data in Supplementary Table S6).

Furthermore, analyses of DE-mRNAs revealed that the key signaling pathways regulating ovine photoperiodic responsiveness and seasonal reproduction, including the thyroid hormone signaling, insulin secretion, Wnt signaling, circadian entrainment, and GnRH signaling pathways were detected after different lengths of photoperiodic treatment (Table 4). For example, compared with LP42, except for *PRKACB* and *PLCB1*, all genes were downregulated in the thyroid hormone signaling pathway, and several genes in circadian entrainment (*PER1*, *RASD1*, and *PRKACB*) were significantly changed with the photoperiod from SP to LP. In addition, with the photoperiod change from SP to LP and gradually from SP-LP3 to SP-LP42, eight photoperiod-related genes, namely, *PRKACB*, *AKT3*, *ATF4*, *KCNJ5*, *CLOCK*, *PLCB1*, *SMAD4*, and *MAPK3*, were upregulated in SP42, and 11 other photoperiod-related genes (*VAMP2*, *BTRC*, *PER1*, *GNB2*, *MTNR1A*, *CDC42*, *ATF4*, *LHB*, *TSHB*, *ESR1*, and *RASD1*) were downregulated. Information on all lncRNAs and their target genes is listed in Supplementary Table S7. In this study, we searched for target protein-coding genes of the differentially expressed lncRNAs to evaluate the potential regulatory functions of lncRNAs, and a total of 130, 75, 132, 101, 145, and 260 target genes of DE-lncRNA were predicted and classified (Supplementary Table S8). Interestingly, we detected some genes related to animal photoperiodic response and estrous seasonality, such as *AANAT*, *TH*, *CAMK2G*, and *KCNJ5*, which were in near to XR_173257.3, XR_173415.3, XR_001024596.2, and XR_001023464.2, respectively (Table 5).

**TABLE 4:** Summary of differentially expressed mRNAs involved in photoperiodic responsiveness and ovine estrous seasonality.

Compared groups	Pathway	DE-mRNAs
SP42 vs. LP42	Thyroid hormone signaling pathway	**PRKACB,** KAT2B, RCAN2, CCND1, NOTCH2, PRKCG, ATP1B1, ATP1A3, and **PLCB1**
	Insulin secretion	**VAMP2, PRKACB,** ATP1A3, **PRKCG,** ATP1B1, PCLO, RAPGEF4, and **PLCB1**
	Wnt signaling pathway	**SMAD4,** DVL1, LRP6, PRKCG, CCND1, **PRKACB, RHOA,** PPP3CB, PRICKLE1, PSEN1, and **PLCB1**
SP42 vs. SP-LP3	Wnt signaling pathway	**SFRP5, RAC1,** DVL1, LRP6, CSNK2A2, **PRKACA,** TBL1X, GSK3B, PRKCG, CSNK2A1, **RHOA, BTRC, and** CTNNBIP1
SP42 vs. SP-LP7	MAPK signaling pathway	HSPA2, CRK, FLNA, MAPK10, RASGRP3, **PRKACB,** RPS6KA3, HSPA8, CACNB3, NF1, DDIT3, CD14, HSPA1L, JUND, GADD45A, **CDC42,** PTPRR, DUSP3, and DUSP7
	Circadian entrainment	GNG11, **PRKACB, KCNJ5, PER1, GNB2, MTNR1A,** GNG4, and GRIA1
SP42 vs. SP-LP15	GnRH signaling pathway	**MAPK13,** PRKACA, ADCY7, ADCY1, **CAMK2D,** CGA, SOS1, **CDC42, ATF4, and LHB**
	Thyroid hormone synthesis	ADCY1, PRKACA, ADCY7, **TSHB, CGA,** ATP1B1, **ATF4, and** ATP1A1
SP42 vs. SP-LP21	Estrogen signaling pathway	GNAO1, HSP70.1, FOS, **AKT3,** CALM3, ATF2, HSPA8, HSP70.1, MMP2, HSPCA, **ATF4,** MAP2K1, **MAPK3,** HSP90AB1, GNAQ, **ESR1, and** NR3A1
	Neurotrophin signaling pathway	RAC1, CRKL, SORT1, **AKT3,** NGFRAP1, MAP2K1, YWHAE, CALM3, ARHGDIA, **CDC42,** MAPK3, **ATF4,** YWHAE, PTPN11, and NGFRAP1
	Circadian entrainment	GNAO1, GRIN2B, FOS, CALM3, GNAQ, **PER1, GNB2, MTNR1A,** MAPK3, and **RASD1**
SP42 vs. SP-LP42	Dopaminergic synapse	MAOB, PRKCG, GRIN2B, PRKCB, GNAO1, PPP1CA, **PRKACB,** CACNA1A, GNAI3, PPP3CC, PPP3CB, GRIA4, **ATF4,** GNG4, CALM3, FOS, GNAI3, CALM3, **AKT3,** PRKACA, **CLOCK,** GSK3A, GNAQ, and PPP2R3C
	Estrogen signaling pathway	CALM3, GNAO1, **AKT3,** FOS, GNAI3, **PRKACB,** MAP2K1, PRKACA, GNAI3, GNAQ, CALM3, HSPA2, SOS1, SHC2, **MAPK3,** HSP90AB1, GABBR2, HSPCA, **ATF4, and** HBEGF
	Circadian entrainment	PRKCB, CALM3, GNAO1, GNG4, CALM3, FOS, **PRKACB,** PRKCG, PRKACA, GRIA4, GNAI3, **PER1,** RPS6KA5, GNAI3, **MAPK3,** GRIN2B, **RASD1, and** GNAQ

**TABLE 5:** Genomic association between lncRNAs and nearby genes related to photoperiodic responsiveness and ovine estrous seasonality.

lncRNAs	lncRNA status	Target mRNAs	Distance	Location
XR_173257.3	Annotated lncRNA	*AANAT*	91, 221	Downstream
XR_173415.3	Annotated lncRNA	*TH*	33, 532	Upstream
XR_001435315.1	Annotated lncRNA	*IGF2BP1*	8, 365	Downstream
		*B4GALNT2 (FecL)*	54, 229	Upstream
XR_001024596.2	Annotated lncRNA	*CAMK2G*	26, 400	Downstream
XR_001434471.1	Annotated lncRNA	*MAPK12*	2, 405	Upstream
LNC_004953	Novel lncRNA	*AKT1*	74, 713	Downstream
XR_001023464.2	Annotated lncRNA	*KCNJ5*	26,151	Downstream

### mRNA–mRNA and lncRNA–mRNA Interaction Networks in Photoperiodic Responsiveness

**FIGURE 8:**
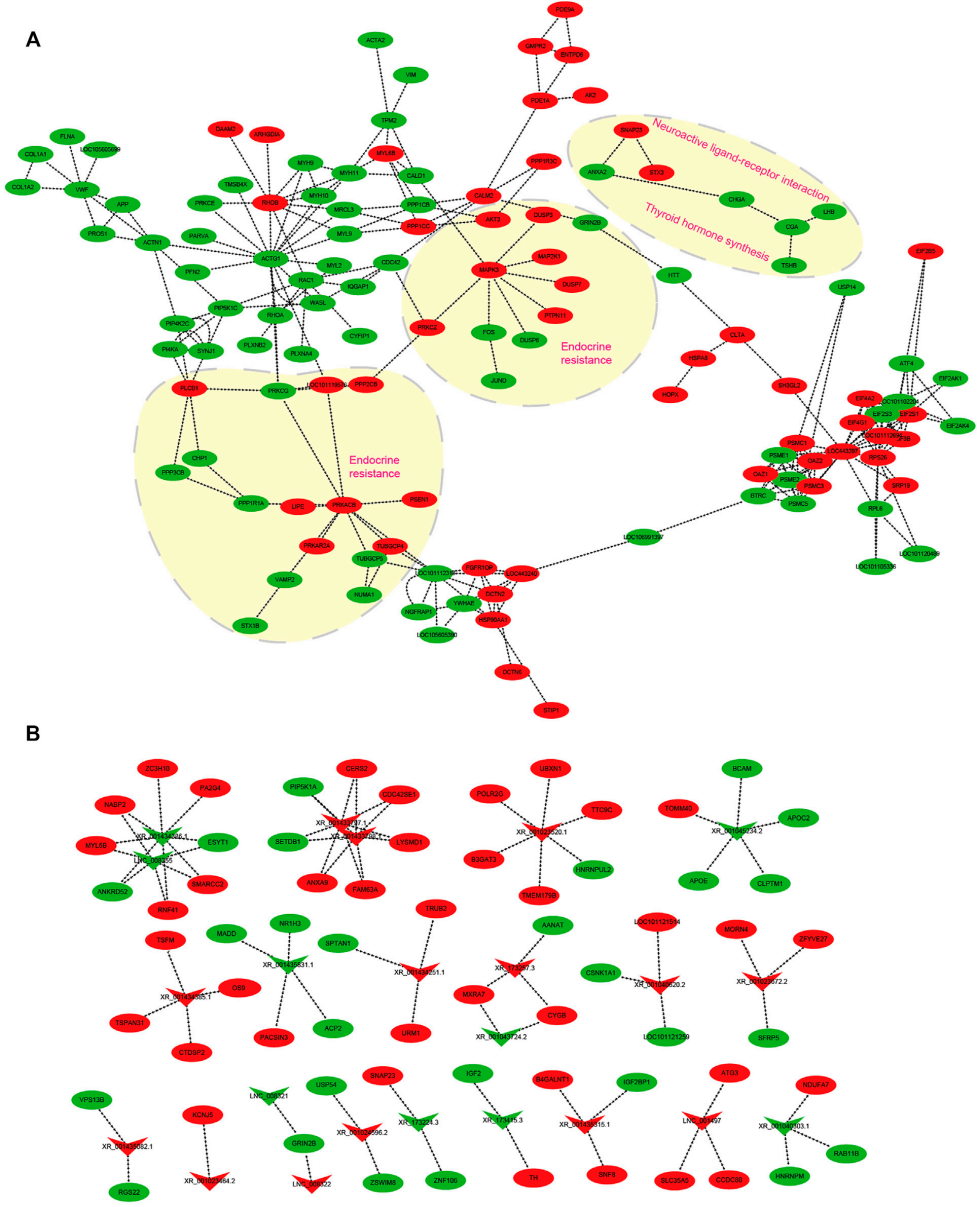
Interaction network in the hypothalamus about ovine photoperiod responsiveness and estrous seasonality. **(A)** mRNA–mRNA interaction network involved in photoperiodic responsiveness, the arrow direction represents the targeting direction; **(B)** lncRNA–mRNA interaction network related to ovine estrus regulation with the photoperiodic change. Circles and “V” represent mRNAs and lncRNAs, red and green represent upregulated and downregulated transcripts, respectively, and the arrow indicates the direction of the target relationship.

Several transcripts were selected to construct mRNA–mRNA and lncRNA–mRNA networks impacted by the change of light from SP to LP (Figure 8). For the mRNA–mRNA interaction network, DE-mRNAs are grouped to insulin secretion (*VAMP2*, *PRKACB*, *PRKCG*, and *PLCB1*), thyroid hormone synthesis (*TSHB*, *CGA*, *ATF4*, and *PRKACA*), and estrogen secretion (*AKT3*, *ATF4*, *MAPK3*, and *ESR1*). DE-mRNAs-regulating hypothalamic hormone secretion and photoperiodic responsiveness were also enriched in GnRH (*MAPK13*, *CAMK2D*, *CGA*, *CDC42*, *ATF4*, and *LHB*), Wnt (*SMAD4*, *RHOA*, *RAC1*, and *SFRP5*) signaling pathways, and circadian entrainment (*PRKACB*, *KCNJ5*, *PER1*, *GNB2*, *MTNR1A*, and *RASD1*) (Figure 8A). Several DE-lncRNAs related to ovine hypothalamic hormone secretion and photoperiodic responsiveness were also selected to construct an lncRNA–mRNA interaction network (Figure 8B). Of these, DE-lncRNAs were highly correlated with steroid hormone biosynthesis, retinol metabolism, and circadian entrainment. The lncRNAs XR_173257.3 targets *AANAT*, and is potentially involved in the response to photoperiodic change. XR_173415.3 targets *TH* and *IGF2*; interestingly, thyroid hormones, encoded by *TH*, have been proven to play an important role in mammalian seasonal reproduction. In addition, XR_001435315.1 and XR_001023464.2 probably participate in ovine reproductive activity because of their target genes *B4GALNT2* (*FecL*) and *KANJ5*.

## Discussion

Mammals exhibit a sensitive photoperiodic response when daylight length reaches a critical boundary (Nishiwaki-Ohkawa and Yoshimura, 2016). Early reports have presented evidence that the sheep hypothalamus plays a vital role in photoperiod-induced reproductive regulation, and that responsiveness to the photoperiod is a key factor that affects ovine puberty onset (Plant, 2015), the estrous cycle transition, and reproductive hormone regulation (Smith, et al., 2007; Smith, 2012; Li, et al., 2019). In previous study, OVX animals have been studied to analyze the mechanism of hormonal feedback, and the location of the major functional area of hypothalamus was in goats (Muñoz, et al., 2017; Liang et al., 2021) and sheep (Goodman, et al., 2011; Weems, et al., 2017; Zhang, et al., 2019; Dardente, et al., 2019); several key genes related to the photoperiodic response, such as *KISS1*, *RFRP*, and *DI O 2/DI O 3*, were identified in these studies. However, few studies have focused on hypothalamic photoperiod regulation at the whole transcriptome level. To explore the effect of photoperiod changes on reproductive hormone regulation and the regulation of hypothalamic gene expression, we established an OVX + E_2_ model in one of the typical seasonal estrus breeds.

### Changes in Reproductive Hormones Under Different Photoperiodic Conditions

Several lines of evidence indicate that there is a cyclical rhythm in ovine reproduction, and its activation and quiescence can be clearly seen in gonadotrophin secretion in OVX + E_2_ ewes (Lincoln, et al., 2006; Menassol, et al., 2012; Dardente, et al., 2019; Guh, et al., 2019). In this study, FSH and PRL were selected as the indicators of the regulation of gonadotropin secretion by photoperiodic changes in OVX + E_2_ ewes. For FSH, the concentration was lowest in the anestrous period and significantly increased in the estrus period; Lincoln (2003) found that the concentration of ovine FSH was very low under LP, which is completely consistent with our findings. This means that the release of FSH was also regulated by photoperiod change or season, and a low concentration of FSH may be related to anestrus in ewes. PRL and FSH display opposite responses to the photoperiod, because they are driven by two different neuroendocrine axes (Dardente, 2012), one or several endocrine factors in the PRL axes have been proved to regulate the photoperiodic rhythms (Lincoln, et al., 2006; Dardente, et al., 2019), our findings also demonstrate that the concentration of PRL increases significantly upon photoperiod extension.

### Differentially Expressed mRNAs in Hypothalamic Photoperiodic Response

In the current study, most omics studies of the hypothalamus have focused on differences in gene expression profiles based on the different estrus periods or some important economic traits with significant differences in animals (Gao, et al., 2018; Hou, et al., 2018; Gley, et al., 2019; Zhang, et al., 2019; Liang, et al., 2021). For example, it was found that the seasonal energy balance of Siberian hamsters was affected by the species-specific responsiveness of hypothalamic T3 (Bao, et al., 2019), and that the hypothalamus was also involved in the circadian of metabolism (Cedernaes, et al., 2019) and daylength response (Sáenz De Miera et al., 2017). Recent studies by *David* emphasized that photoperiodic transitions in endocrine output are regulated by a multistep signaling cascade within the mammalian neuroendocrine system (Hazlerigg, et al., 2018), and the differences of hypothalamic transcriptome levels can provide more information about photoperiodic response in ewes. In this study, compared with SP42, most DE-mRNAs in the SP-LP3 to SP-LP42 groups compared with the SP groups were associated with the signaling of circadian entrainment, insulin secretion, thyroid hormone, and estrogen (Table 4). As previously reported, SCN lesions lead to the alterations in the estrous cycle, sexual behavior, and tonic and phasic secretion of GnRH and gonadotropins in rodents, and it has been proven that *Clock* and *Clock*-related genes are the key factors in mammalian reproductive cycles (Silva and Domínguez, 2019). In our studies, the expression of *Clock*-related genes, such as *MTNR1A*, *PER1*, *DI O 2*, and *ESR1*, was changed with the artificial photoperiod from SP42 to LP21 and LP42, and was synchronously downregulated with gonadotropins FSH. Importantly, previous studies have demonstrated that these genes were significantly correlated with reproductive seasonality in goats and sheep (Wood, 2018; He, et al., 2019). Astoundingly, hypothalamus mRNAs that were altered in the SP–LP groups (SP-LP7, SP-LP21, and SP-LP42) compared to the SP42 group were all associated with circadian entrainment (Table 4), which was proven to initiate the maturation of human islets and insulin secretion (Alvarez-Dominguez, et al., 2019). Moreover, recent evidence has shown that insulin plays a vital role in the arrangement of reproductive activities through direct actions on insulin receptors; coincidentally, these receptors are present in several areas that are well-known to play a crucial role in reproduction, such as the ARC, ventromedial hypothalamic nucleus (VMH), and preoptic area (POA) (Caraty, et al., 1998; Brüning, et al., 2000; Cernea, et al., 2016). Several genes that were significantly differentially expressed upon photoperiod changes in our studies participate in insulin secretion (*VAMP2*, *PRKACB*, and *PLCB1*) and circadian entrainment (*PRKACB*, *RASD1*, and *KCNJ5*), and the change of previously mentioned key genes in circadian entrainment triggers insulin secretion; subsequently, the receptors in various hypothalamic functional areas are activated to regulate the ovine photoperiod-induced reproductive activities.

### LncRNAs and Functional Pathways Related to Photoperiodic Response

At present, there is vast availability of evidence suggesting that the expression of lncRNAs can show high correlations with the expression of neighboring mRNAs. As stated in the introduction, lncRNA GnRH-E1 RNA targets its nearby gene GnRH1 to contribute to the regulation of GnRH neuronal maturation (Huang, et al., 2016). Furthermore, *XLOC_1041225* and *XLOC_446331* target neighboring *IGFBP5*, and regulate the onset of puberty in goats (Gao, et al., 2018). The ovine hypothalamic lncRNAs found in the present study had many similar characteristics with those of other mammals, such as shorter and fewer exons, shorter ORFs, and lower expression levels than protein-coding transcripts (Figure 4). As discussed in 4.3, genes involved in circadian entrainment and insulin secretion participate in photoperiod-induced reproduction in sheep. Several lncRNAs target genes were associated with the signaling of circadian entrainment (*KCNJ5* and *CAMK2G*), prolactin (*TH* and *AKT1*), steroid hormone biosynthesis (*UGT1A1*), and tryptophan metabolism (*AANAT*) (Table 5 and Figure 6). As previously reported, the *AANAT* gene is an essential enzyme for the rhythmic synthesis of melatonin (Maronde, et al., 2011), and *TH* can also be connected with *AANAT* using dopa decarboxylase (*DDC*). Combined with the earlier studies, our results indicated that the *XR_173257.3*-*AANAT* and *XR_173415.3*-*TH* networks may be pivotal in rhythmic melatonin synthesis and secretion and subsequent reproductive seasonality in sheep. Moreover, *KCNJ5*, also known as a G protein-activated inward rectifier potassium channel 4 (*GIRK4*), has been confirmed to be associated with circadian rhythmicity in humans (Hsiao, et al., 2019). From our results, *XR_001023464.2-KCNJ5* synchronous expression may be involved in the ovine circadian rhythm induced by photoperiod changes. Interestingly, we also detected the differentially expressed lncRNA *XR_001435315.1*, in which the target gene *B4GALNT2* was proven to be associated with the ovine litter size in our earlier study (Guo, et al., 2018). All these findings suggest that lncRNAs may be a regulator involved in the photoperiodic response in sheep.

## Conclusion

In conclusion, reproductive hormone detection and transcriptome analysis are useful for understanding the effects of photoperiod change on reproductive seasonality in ewes. Our studies, for the first time, identified pivotal changes in reproductive hormone levels and transcriptomic expression with photoperiod changes in OVX + E_2_ ewes. FSH and PRL concentrations showed significant changes with the photoperiod. Moreover, we suggest that the transcriptome levels change to coordinate with circadian rhythms. We identified major genes (such as *VAMP2*, *PRKACB*, *PLCB1*, *RASD1*, and *KCNJ5*) and lncRNAs (such as *XR_173257.3*, *XR_173415.3*, and *XR_001023464.2*) as candidate transcripts for photoperiod responses, and these factors may interact to regulate hormonal changes and gene expression through the enriched pathways of insulin secretion, circadian entrainment, and thyroid hormone signaling. These findings proved that the application of OVX + E_2_ is important for the study of photoperiodic effects in ewes; simultaneously, the differences in reproductive hormones and the transcriptome provide a valuable resource for further study of the molecular mechanisms of the hypothalamic-regulated reproductive seasonality in ewes.

## Data Availability

The datasets presented in this study can be found in online repositories. The names of the repository/repositories and accession number(s) can be found in the article/Supplementary Material.
