# Supplementary figures and images for: Transcriptomic Changes of Photoperiodic Response in the Hypothalamus Were Identified in Ovariectomized and Estradiol-Treated Sheep
Source: Front Mol Biosci. 2022 Apr 11;9:848144. doi: 10.3389/fmolb.2022.848144 (PMC9036065; doi:10.3389/fmolb.2022.848144)

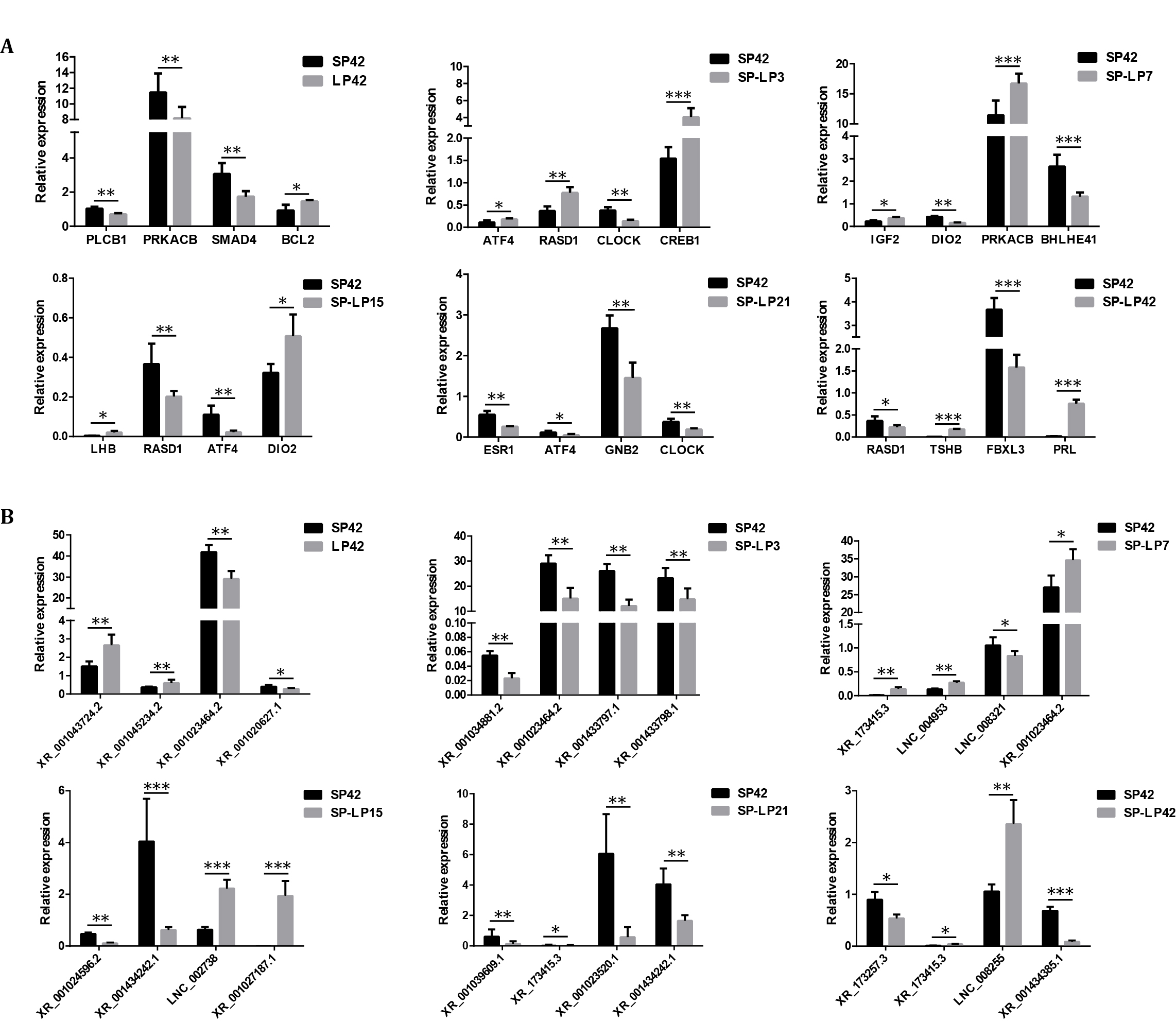

Supplement: Supplementary file 3 [file Image1.JPEG]
